# Editorial: Uncovering physiological mechanisms of aquatic organisms under environmental stress in a changing climate

**DOI:** 10.3389/fphys.2026.1960627

**Published:** 2026-08-14

**Authors:** Analía Ale

**Affiliations:** Cátedra de Toxicología, Farmacología y Bioquímica Legal (FBCB-UNL), CONICET, Santa Fe, Argentina

**Keywords:** aquatic physiology, biomarkers, climate change, environmental stressors, multilevel biological response, physiological adaptation, pollution impacts

## Introduction

Aquatic ecosystems are being reshaped by climate change faster than most predictive frameworks can accommodate. Warming, marine heatwaves, cold shocks, acidification and altered hydrology do not act on otherwise undisturbed organisms: they act on populations already coping with chemical pollution, habitat degradation and shifting food webs. Whether a species persists under these conditions is ultimately a physiological question — it depends on the cellular, metabolic and molecular machinery that determines how much stress an organism can absorb, for how long, and at what cost. This Research Topic was conceived to bring that mechanistic layer to the foreground, moving beyond descriptions of where and how much impact occurs towards an understanding of why particular organisms fail while others resist. The seven contributions gathered here span marine and freshwater systems, invertebrates, fish, to cetaceans, and controlled experiments to multi-stressor syntheses. They differ in taxa, stressors and methodological scale, yet they share a common analytical stance: physiological and molecular endpoints are treated not as endpoints in themselves, but as the mechanistic bridge between an environmental driver and its ecological consequence.

## Mechanistic evidence from marine systems

Marine systems dominate this Research Topic, with five experimental studies spanning contrasting taxa and stressors. Liu et al. examined acute cold stress in juvenile yellowfin tuna (*Thunnus albacares*), uncovering divergent antioxidant, metabolic and transcriptional trajectories in red and white muscle: antioxidant activities peaked early and then declined, while red muscle sustained induction of *hspa1b* and *acadm* and white muscle responded faster but proved more vulnerable to damage. Spencer et al. contrasted 8-hour and 88-day transcriptomic responses of juvenile snow crab (*Chionoecetes opilio*) to ocean acidification, separating short-term tolerance from chronic stress. The early response mobilized mitochondrial repair, cuticle maintenance and immune modulation; after 88 days, however, sustained upregulation of damage-mitigation pathways under severe acidification revealed a chronic stress state that preceded any outward physiological effect and anticipated later mortality. Their identification of a putative carbonic anhydrase 7 gene as a candidate biomarker illustrates the practical payoff of such designs.

Two further contributions show that climate impacts often reach organisms indirectly, and that ontogeny governs who is exposed to the greatest risk. Silva et al. mapped critical thermal maxima across the life cycle of the stenophagous nudibranch *Berghia stephanieae*, finding juveniles to be the least tolerant stage, thermal tolerance plasticity to be minimal, and embryos to fail under prolonged heatwave conditions. In a companion study, Silva et al. showed that a marine heatwave combined with a diet of bleached prey impaired the cellular stress response, reduced egg output and lowered survival; a performance breakdown driven as much by what the animal ate as by temperature itself. Specialized consumers, rarely included in climate vulnerability assessments, may therefore pay a disproportionate toll.

Finally, Mantor et al. confront a persistent constraint: the species of greatest conservation concern are often the least tractable experimentally. Using precision-cut adipose tissue slices from humpback whale blubber, they showed that ex vivo cortisol exposure, alone or combined with epinephrine, upregulated *PPARG* and downregulated *TNF* and *TLR4*, indicating that stress hormones drive blubber towards an anti-inflammatory state with potential immune consequences. Beyond the finding itself, the approach offers a non-lethal, reduction-aligned platform for probing multiple stressors in free-living cetaceans.

## Freshwater perspectives: mixtures as the realistic scenario

The two freshwater contributions are reviews, and both converge on the same diagnosis from different starting points: single-stressor designs no longer describe the conditions organisms actually face. Ale et al. synthesized nanoplastic ecotoxicity under pollutant co-exposure and climate-derived stressors, finding synergism (frequently mediated by a Trojan horse mechanism) to be the dominant reported interaction, and rising temperature to amplify nanoplastic toxicity across taxonomic groups. The available evidence, however, rests heavily on *Chlorella* spp., *Daphnia magna* and *Danio rerio*, leaving molluscs and macrocrustaceans as critical blind spots under climate change scenarios. Wickramasinghe and Ruvinda reach a parallel conclusion regionally, reviewing morphological, physiological and behavioral biomarker responses in Sri Lankan lotic fishes exposed to complex pollutant mixtures, and identifying the interplay between emerging contaminants (nanoparticles, microplastics, pharmaceuticals and endocrine disruptors) and climate-related physicochemical variation as the principal gap for indigenous species. Both reviews underscore that inland waters concentrate contaminant loads while offering organisms fewer thermal refuges than open marine systems, so interactive effects are likely to be both stronger and less easily escaped.

## Emerging priorities

Read together, these contributions point to three priorities. First, temporal design deserves explicit justification. Tolerance measured over hours is not tolerance: both Liu et al. and Spencer et al. show that conclusions invert depending on the exposure window, and studies should state the timescales over which their inferences hold. Second, early-warning biomarkers need cross-taxon validation. Several contributions independently converge on molecular signatures that precede organismal failure; linking these to survival and reproductive endpoints would convert them from descriptive readouts into usable tools for risk assessment. Third, taxonomic and geographic representation must be widened. Model species dominate, ecologically relevant taxa remain largely untested under realistic co-exposure, and tropical and Southern Hemisphere freshwater systems are conspicuously underrepresented.

Meeting these challenges will require multi-stressor experimental frameworks built on environmentally realistic concentrations and climate scenarios, coupled with predictive approaches capable of anticipating interaction outcomes rather than documenting them case by case. The physiological mechanisms explored here are, ultimately, the currency in which climate change is paid. We thank all authors and reviewers whose work made this Research Topic possible.

